# Characteristics of young people accessing recently implemented Community Forensic Child and Adolescent Mental Health Services (F:CAMHS) in England: insights from national service activity data

**DOI:** 10.1007/s00787-021-01870-y

**Published:** 2021-09-14

**Authors:** Rebecca Lane, Sophie D’Souza, Rosie Singleton, Nick Hindley, Dickon Bevington, Oliver White, Jenna Jacob, James Wheeler, Julian Edbrooke-Childs

**Affiliations:** 1 Institute of Psychiatry, Psychology and Neuroscience, King’s College London, 16 De Crespigny Park, Camberwell, London, SE5 8AB UK; 2 Anna Freud Centre, The Kantor Centre of Excellence, 4-8 Rodney Street, London, N1 9JH UK; 3 University College London, Gower Street, London, WC1E 6BT UK; 4 Anna Freud Centre, Child Outcomes Research Consortium, Kantor Centre for Excellence, 4-8 Rodney Street, London, N1 9JH UK; 5 South Central F:CAMHS, Oxford Health NHS Foundation Trust, Maple House, the Slade, Horspath Driftway, Oxford, OX3 7JH UK; 6 University of Oxford, Oxford, OX1 2JD UK; 7 NHS England and NHS Improvement, Skipton House, 90 London Road, London, SE1 6LH UK; 8 Southern Health NHS Foundation Trust, Tatchbury Mount, Calmore, Southampton, SO40 2RZ UK

**Keywords:** Forensic CAMHS, Mental health, Forensic psychiatry, Child and adolescent psychiatry

## Abstract

Children and young people in contact with forensic child and adolescent mental health services present with more complex needs than young people in the general population. Recent policy in child and adolescent mental health has led to the implementation of new workstreams and programmes to improve service provision. This research examines the characteristics of children and young people referred to recently commissioned Community Forensic Child and Adolescent Services (F:CAMHS) and service activity during the first 24 months of service. The study is a national cohort study to describe the population and investigate service provision and access across England. Secondary data on 1311 advice cases and 1406 referrals are included in analysis. Findings show that 71.9% of the sample had accessed mainstream CAMHS before their referral, 50.9% had experienced/witnessed multiple traumatic events and 58.4% of young people presented with multiple difficulties. The results of the study highlight the complexity of the cohort and a need for interagency trauma-informed working. This is the first study to describe the characteristics of children and young people referred to Community F:CAMHS and provides valuable information on pathways and needs to inform service policy and provision.

**Supplementary Information:**

The online version contains supplementary material available at 10.1007/s00787-021-01870-y.

## Introduction

A wealth of research highlights the increased prevalence of mental health difficulties including multimorbid and complex difficulties, higher levels of psychosocial adversity, learning difficulties, and substance misuse difficulties in young people who present with high risk, compared with their peers in the general population [1–3]. The term ‘high risk’ is used to refer to young people about whom there are concerns about risk of harm to others and who may be in contact with the youth justice system, as well as to denote young people who are vulnerable in other ways, including being at risk of self-harm or exploitation. For some very vulnerable children and young people, particular mental health needs may be difficult to meet through conventional services due to their unique and complex circumstances [4]. Furthermore, children and young people who present with high risk of harm to others or to self have commonly not been in contact with mainstream Child and Adolescent Mental Health Services (CAMHS), despite having high rates of mental health difficulties [5]. This group of children and young people are subject to frequent transitions between services and geographical displacement and have been traditionally underserved and stigmatised by services, thus exacerbating the difficulty building trusting relationships with professionals, as well as the system at large. Previous research [6] highlights that service provision for young people with higher levels of risk across agencies has previously been fragmented and lacking in coordination. Social disadvantage and the barriers to accessing support place young people who present with high risk of harm to others or to self at further risk of behaviour difficulties and social isolation, which can have a considerable negative impact on life chances [7].

Child and adolescent mental health policy, such as Future in Mind [8] and the Five Year Forward View [4], identifies a need to improve mental health care and support for children and young people and has emphasised the need to prioritise mental health research and greater parity of provision. As part of the response to such recommendations, 13 regional Community Forensic Child and Adolescent Mental Health Services (F:CAMHS) were nationally commissioned in 2017/2018 to address previous gaps in service provision and offer coverage across England. These services may include staff from a variety of professional groups including, but not limited to: psychiatrists, clinical and forensic psychologists, nurses, social workers, and other allied disciplines [6].

Community F:CAMHS are targeted towards children and young people with complex needs and high-risk presentations (including behaviour difficulties) about whom professionals are concerned [6, 9]. Referrals are accepted if those who are referred meet the above target group and/or there is a need for expert input as a result of significant professional concern, for instance due to safeguarding. The services have been created to support professional networks and assist colleagues in a range of agencies working with children and young people who present with high levels of concern and complexity, with a maximum initial response time of five working days. The services also aim to provide advice about the interactions between a child or young person’s mental health and risk presentation and to improve pathways and transfers between local and national services, which include secure mental health, welfare and youth justice settings as well maintaining contact with young people placed elsewhere away from home [8]. Community F:CAMHS offer the opportunity for both indirect and direct interventions. ‘Indirect’ interventions involve the provision of informed support to professionals who contact the service with concern and uncertainty about a young person. Such provision can involve (a) an initial short informal advisory discussion resulting in a decision about whether further service involvement is required or (b) more formal professional consultation with a single professional or with a wider professional network. ‘Direct’ interventions involve the face-to-face involvement of Community F:CAMHS in clinical work, assessment and ongoing clinical input with a young person when this is considered necessary. All these functions form part of the Community F:CAMHS service model with case status being recognised in contacts which proceed to either formal consultation or direct clinical input [9].

This is a new national approach to the provision of forensic support for mainstream CAMHS and other services, designed to adapt the support provided according to the needs of, and resources available to both young people *and* services. A spectrum of interventions is possible that begin by scaffolding existing services through consultation or assessment and advice, but where required, can provide more intensive face-to-face interactions, and if necessary, over longer timescales. This privileging and scaffolding of *existing working therapeutic/helping relationships* in the community is intended to resist the fragmenting tendency of systems.

The national Community F:CAMHS service specification is modelled on previously existing, smaller-scaled services. Evaluations of these services in the Thames Valley and Hampshire and Isle of White regions in the UK were conducted using activity data and feedback from professionals likely to refer to Community F:CAMHS [5, 10]. The results of the evaluations suggested that Community F:CAMHS was highly valued in the professional community, with one commissioner noting a reduction in referrals for out-of-area forensic assessments, avoiding displacement and additional costs [5]. Professionals also reported that Community F:CAMHS decreased the potential for vulnerable young people to fall through gaps between services [10].

These small-scale evaluations also provided initial insights into the characteristics of young people accessing Community F:CAMHS and service activity. Out of a total of 278 referrals, the largest proportion of young people referred were male (82%), aged between 13 and 15 years (44%), White British (65%), and referred from mainstream CAMHS (52%) [6]. The evaluations provided sufficient evidence of promise about the Community F:CAMHS model to enable some regional commissioning arrangements in Southern England to be provided on a longer-term basis but they were far from representative of national needs and geographical and social variations. Importantly, the evaluations provided little detailed information on children and young people’s characteristics beyond baseline demographic information. Further research is needed to explore who is referred to Community F:CAMHS nationally to better understand the needs and complexities of children and young people who present with high-risk.

### Rationale and aims

The present study aims to meet the aforementioned gaps in understanding. As Community F:CAMHS teams have now been implemented within England there is an opportunity to evaluate service activity and the characteristics and outcomes of young people accessing the services at a national level. Evidence is needed to: (a) collate a national picture of service activity and (b) examine the characteristics of children and young people accessing Community F:CAMHS. This study aims to address these gaps by examining routine service activity data from 13 Community F:CAMHS during their first two years of service delivery.

## Methods

### Study design

The current research is part of a national cohort study by the Anna Freud National Centre for Children and Families (AFNCCF) evaluating Community F:CAMHS funded by NHS England and NHS Improvement (NHSE&I) between March 2018 and March 2021 [11]. Routine service activity data collected by the 13 Community F:CAMHS teams between April 2017 and January 2020 are included in the current study. The majority of the data provided were collected from referral forms and clinical entries. Community F:CAMHS teams were trained by RL and SD to use a template for data submissions and were provided with data dictionaries and guidance sheets. Data were submitted directly and securely to the research team by individual services and were subject to a validation process.

### Materials

For the purposes of data collection, the service activity data collected were divided according to the type of contact made with Community F:CAMHS and whether it represented (1) informal advice and discussion (2) formal referrals resulting in indirect (formal consultation) or direct work (direct assessment and direct intervention).

### Informal Community F:CAMHS involvement: advice

Advice refers to cases where informal advice is given in response to an initial contact made either by phone or email to Community F:CAMHS. The F:CAMHS teams are accessible to professionals from all agencies and disciplines within geographical catchment for such advice. Outcomes from an advice call include recommendations that a formal referral should be made to the team and other alternatives agreed with the professional in question. Data about advice activity are collected by Community F:CAMHS teams and is less comprehensive than that available from formal service referrals; such data comprises only age, gender, and contact source.

### Formal Community F:CAMHS involvement: consultation and direct clinical involvement

Formal Community F:CAMHS cases are those that are formally referred, enter the Community F:CAMHS caseload, and receive indirect and/or direct input from the service. Data in these cases are collected by Community F:CAMHS and include information from a structured referral form resulting in a more extensive data set. The data collected includes information on service activity, such as referral source, reason for referral, and Community F:CAMHS input with corresponding background information including family background and relationships, trauma history and presenting difficulties. Please note that these presenting difficulties do not necessarily imply formal diagnoses and instead reflect clinical judgement at referral.

Data collection was organised to allow for further data to be collected where more extensive Community F:CAMHS input was required. Variables were categorised as either ‘essential’ or ‘desirable’. Missing data are termed ‘Not known/not provided’ to account for desirable data which may not have been prioritised for submission. A high proportion of ‘Not known/not provided’ data is present in the dataset. Across all variables relevant to referral cases included in this paper, ‘Not known/not provided’ data per case ranged from 3.8 to 97.1%, mean = 24.9%. For variables termed essential (see “Methods” section), not known/not provided data per case ranged from 1.7 to 94.8%, mean = 8.9%.

Prior to data analysis, some variables were collapsed for ease of reporting and low frequency variables were additionally generated, combining variables with a prevalence of < 5% (i.e. < 5% ‘yes’). Low frequency variables are coded as ‘yes’, if yes was present for any of the combined variables, ‘no’ if all combined variables were no and otherwise missing. See Tables below for more information.

### Participants

All children and young people accessing Community F:CAMHS, whether through advice or referring channels, were eligible for inclusion if consent to share data was granted to local services and necessary data sharing agreements were in place.

### Ethical considerations

Ethical approval for data collection was granted by the Health Research Authority (18/LO/1569). Routine service data were shared in line with data sharing agreements and according to local consent procedures. Data were pseudonymised prior to submission, removing identifiable information such as names or National Health Service (NHS) numbers. Special Category Data, as defined in the General Data Protection Regulation (EU 2016/679) (GDPR), including ethnicity and medical history (i.e., routine service data), was only shared if the above requirements were met. All electronic data were stored using the secure information governance infrastructure at University College London in compliance with the Data Protection Act (2018), GDPR [EU 2016/679)] and ISO27001.

### Analysis

A description of national activity will be presented using the anonymised routine service data. The characteristics of the cases will be further explored using descriptive statistics. Although the focus of the research is to describe the characteristics of young people in contact with Community F:CAMHS, significance testing will also be performed on select groups to enhance understanding of the relation between service activity and case characteristics. These groups are: gender, previous access to CAMHS, and contact type. All analyses will be conducted using R version 3.6.2 [12].

## Results

### Key findings from the significance tests are presented in the results section, and results of all tests are presented in supplementary tables service activity

#### Sources of requests for advice or referrals

Overall, data on 2717 cases were received from all 13 Community F:CAMHS: 48.3% (*n* = 1311) advice cases and 51.7% (*n* = 1406) referral cases. The most common contact/referral source for all cases was mainstream CAMHS (46.0%, *n* = 1250), and this remained the most common contact/referral source for both advice and referral cases. Other contact/referral sources include 23.0% from social care (*n* = 625), 15.1% from youth justice (*n* = 409), 6.92% from education (*n* = 188) and 0.81% from General Practitioners (GPs) (*n* = 22); see Table 1.

**Table 1:** Contact/referral source and reason for referral

	All cases		Advice		Formal referrals	
	*N*	%	*N*	%	*N*	%
Age (years)						
8 or younger	95	3.5	57	4.4	38	2.7
9–11	229	8.4	115	8.8	114	8.1
12–15	1324	48.7	591	45.1	733	52.1
16 or older	978	36.0	464	35.4	514	36.6
Not known/not provided	91	3.4	84	6.4	7	0.5
Contact/referral source						
Mainstream CAMHS	1250	46.0	644	49.1	606	43.1
Social care	625	23.0	260	19.8	365	26.0
Youth justice	409	15.1	199	15.2	210	14.9
Education	188	6.9	102	7.8	86	6.1
Other health	105	3.9	49	3.7	56	4.0
GP	22	0.8	9	0.7	13	0.9
Third sector	14	0.5	6	0.5	8	0.6
Other	64	2.4	40	3.1	24	1.7
Not known/not provided	40	1.5	SN^a^	–	38	2.7
Reason for referral						
Violence/aggression						
Yes					1125	80.0
No					215	15.3
Not known/not provided					66	4.7
Second opinion in complex case						
Yes					434	30.9
No					904	64.3
Not known/not provided					68	4.8
Sexually harmful behaviour						
Yes					424	30.2
No					916	65.1
Not known/not provided					66	4.7
Criminal justice						
Yes					349	24.8
No					989	70.3
Not known/not provided					68	4.8
Fire setting						
Yes					147	10.5
No					1192	84.8
Not known/not provided					67	4.8
Other^b^						
Yes					233	16.6
No					1094	77.8
Not known/not provided					79	5.6

^a^SN—small numbers (< 3) are not reported to protect anonymity, data may also not be reported to prevent calculation of small numbers

^b^‘Other’ referral reasons included: parasuicidal behaviour, animal cruelty, gang affiliation, psychosis, risk of child criminal exploitation

For 65.1% of the 1406 cases, referral to Community F:CAMHS led to indirect case input (*n* = 915), 25.9% to direct case input (*n* = 364), 1.9% (*n* = 27) to other input types (these included provision of written advice and direct input to family). Of all referrals, 4.6% were rejected (*n* = 65), and for 2.5 referrals this information was not known/not provided (*n* = 35).

The age of advice and referral cases is presented in Table 1. A higher proportion of younger children and young people are advice-only cases, 4.4% were aged 8 or younger, compared to 2.7% of formal referrals (See Table 1). A *χ*^2^ test of independence was performed to examine the relationship between age and Community F:CAMHS input (direct or indirect). This was significant, *χ*^2^ (3, *N* = 1279) = 11.32, *p* < 0.05, with more children and young people in the younger age ranges receiving indirect compared to direct input.

#### Reasons for referral

Overall, 80.0% of referrals were for violence/aggression (*n* = 1125), 30.2% for sexually harmful behaviour (*n* = 424), and 30.9% for a second opinion in complex case (*n* = 434), as shown in Table 1. Second opinion in complex case refers to a case involving major complexity, with or without legal issues, where there are some concerns about risk and not meeting other criteria. Reasons for referral were not mutually exclusive, with 37.1% (*n* = 522) cases presenting with two referral reasons and 26.5% (*n* = 373) cases with three or more. A *χ*^2^ test of independence was performed to examine the relation between referral reason and Community F:CAMHS input. The relation between these variables was significant for criminal justice, *χ*^2^ (1, *N* = 1279) = 4.90, *p* < 0.05, and for second opinion in complex case, *χ*^2^ (1, *N* = 1279) = 10.20, *p* < 0.01, with young people more likely to receive direct input and indirect input, respectively. No other significant differences were found.

#### Types of intervention

For all referral cases, 48.2% required multi-agency case management (*n* = 677) and 48.4% required ongoing indirect monitoring (*n* = 681). Types of Community F:CAMHS intervention was not mutually exclusive, with 25.2% (*n* = 354) cases with two types of intervention and 15.7% (*n* = 221) with three or more.

#### Agencies involved and Community F:CAMHS input duration

At time of referral, mainstream CAMHS were involved in the care of 63.4% (*n* = 892) children and young people referred to Community F:CAMHS. Nationally, 71.9% (*n* = 1011) of children and young people had accessed mainstream CAMHS before referral to Community F:CAMHS, ranging from 64.6 (East) to 80% (South). A *χ*^2^ test of independence showed a significant difference in referral source for children and young people who accessed mainstream CAMHS before referral and those who had not, *χ*^2^ (8, *N* = 1406) = 95.413, *p* < 0.001. In contrast to the national picture, the most common referral source for the subsample of children and young people who had not accessed mainstream CAMHS before referral was Social Care (35.6%; *n* = 125). Furthermore, 6.6% (*n* = 93) of all young people had been known to Community F:CAMHS prior to referral.

Of referrals, 60.2% of cases (*n* = 847) were discharged from Community F:CAMHS as of January 2020. The mean duration of Community F:CAMHS input (time from referral date to discharge date) was 116 days (range = 0–737). Some cases required longer term Community F:CAMHS input. Of all discharged and open cases, 5.5% of cases (*n* = 76) required longer term Community F:CAMHS input, greater than one year. For open cases, duration of Community F:CAMHS input was estimated using time from referral date to last discharge or referral date reported by the team. In 14.5% of discharged cases (*n* = 123), there had been an escalation in support during Community F:CAMHS involvement. Escalation in support could refer to transition from indirect to direct involvement or transition from single to multi-agency working during Community F:CAMHS involvement in a case.

### Characteristics and background of young people accessing Community F:CAMHS

For the 2717 children and young people who accessed Community F:CAMHS, 80.8% (*n* = 2194) were male (includes trans male) and the mean age for the sample was 14.3 years (sd = 2.51; range 3–21).

#### Advice cases

Data on 1311 advice cases were collected by 11 Community F:CAMHS. 78.9% (*n* = 1034) of advice cases were male and the mean age was 14.24 years (sd = 2.7 years, range = 3–21). No other data on characteristics and background were collected for advice cases.

#### Referral cases

Data on 1406 referrals were collected by 13 Community F:CAMHS. 82.5% (*n* = 1160) of referral cases were male and the mean age was 14.3 years (sd = 2.33, range = 5–18). 76.9% of young people referred to Community F:CAMHS were from a White ethnic background (*n* = 1081), see Table 2. The ethnic background of cases varied across regions, from 47.0% of cases (*n* = 109) being from a White ethnic background in London to 85.9% (*n* = 292) in the North of England (see Appendix). A *χ*^2^ test of independence was performed to examine the relations between access to mainstream CAMHS prior to referral and gender with ethnicity, finding no significant differences between groups.

**Table 2:** Characteristics of young people referred to Community F:CAMHS

	All cases	
	*N*	%
Ethnicity		
White	1081	76.9
Mixed	123	8.8
Black/Black British	77	5.5
Asian/Asian British	40	2.8
Other ethnic group^a^	29	2.1
Not known/not provided	56	4.0
Social care status^b^		
Social care or early help plan	499	35.5
Looked after children	423	30.1
No social care involvement	326	23.2
Other	108	7.7
Not known/not provided	50	3.6
Youth justice status^c^		
Recent police contact	318	22.6
Sentenced	190	13.5
Pre-sentencing	112	8.0
Other	84	6.0
Not applicable	657	46.7
Not known/not provided	45	3.2
Education status^d^		
Specialist provision	540	38.5
Mainstream	375	26.7
NEET	275	19.6
Further education/employment	45	3.2
Other	127	9.0
Not known/not provided	44	3.1
Living arrangement^e^		
Family	828	58.9
Supported residential status	330	23.4
Secure accommodation	68	4.9
Adoptive family	58	4.1
Inpatient unit	53	3.8
Independent living	4	0.3
Other	30	2.1
Not known/not provided	35	2.4

A number of variables were collapsed for ease of reporting. If not listed below, the variable displayed is in its original form

^a^‘Other ethnic group’ included Afghani, Ethiopian, Iraqi, Iranian and Turkish

^b^Social Care Status: ‘Looked After Children’ includes ‘Leaving Care’, ‘Looked After S20’, ‘Looked After S31’ and ‘Secure Accommodation Order’; ‘Social Care or Early Help Plans’ includes ‘Child in Need’, ‘Subject to CP Plan’, ‘Team Around the Child’ and ‘Allocated Social Worker’

^c^Youth Justice Status: ‘Sentenced’ includes ‘Sentenced—community order’ and ‘Sentenced—custodial’; ‘Pre-sentencing’ includes ‘On bail’, ‘On remand’ and ‘Pre-court order’

^d^Education Status: ‘Further Education/Employment’ includes ‘College of Further Education’, ‘Vocational Training’ and ‘Left School (employed)’; ‘Specialist provision’ includes ‘Mainstream SEN’, ‘SEN’, ‘Home Tuition’, ‘Special School’, ‘Pupil Referral Unit’ and ‘ Hospital School’

^e^Living Arrangement: ‘Family’ includes ‘Birth Family’ and ‘Other Family’; ‘Supported residential status’ includes ‘Foster Care’, ‘Semi-independent Living’, ‘Residential Care’, and ‘Residential School’; ‘Secure Accommodation’ includes ‘YOI’, ‘Secure Care (CJS)’ and ‘Secure Care (Welfare)’; ‘Inpatient Unit’ includes ‘PICU’, ‘Low/Medium Inpatient Secure’ and ‘Open Inpatient Unit’

#### Young people’s social care, educational, youth justice, and residential status

Social Care status, Education status, Youth Justice status and Living Arrangement at referral for Community F:CAMHS cases are presented in Table 2. In particular, 35.5% (*n* = 499) of children and young people referred had Social Care or Early Help Plans. Youth Justice status was not applicable for 46.7% (*n* = 657) of children and young people referred, and 22.6% (*n* = 318) of children and young people had recent police contact. Moreover, 38.5% (*n* = 540) of children and young people referred to Community F:CAMHS were accessing specialist provision for their education at referral and 19.6% (*n* = 275) were not in education, employment or training. At referral, 23.4% (*n* = 330) of children and young people held supported residential status, including residential school and foster care. Furthermore, 8.7 (*n* = 122) of children and young people referred were currently placed or living out of area. A *χ*^2^ test of independence was performed to examine the relationship between gender and social care status and youth justice status. The relation was significant between gender and social care status *χ*^2^ (4, *N* = 1398) = 23.716, *p* < 0.0001. A greater proportion of females  are looked after children (see Supplementary table 2). A *χ*^2^ test of independence was also performed to examine the relationship between gender and referral source, and referral reasons. The relationship between gender and reason for referral-youth justice was significant *χ*^2^ (1, *N* = 1398) = 9.5608, *p* < 0.01. The relationship between gender and referral source was also significant *χ*^2^ (1, *N* = 1398) = 22.388, *p* < 0.01. A higher proportion of males had youth justice as a referral reason and were referred by youth justice than females. A greater proportion of females in contact with Community F:CAMHS were referred by mainstream CAMHS than males (see Supplementary Table 2).

#### Young people’s mental health and wellbeing presentation at referral

Children and young people referred to Community F:CAMHS presented with a range of mental health and wellbeing difficulties; see Table 3. Overall, 79.3% (*n* = 1116) of all children and young people presented with at least one difficulty related to psychosis, anxiety, depression, post-traumatic features, attention deficit and hyperactivity disorder, autism, and conduct and longstanding behaviour difficulties. These were not mutually exclusive, with 58.4% (*n* = 821) of children and young people having two presenting difficulties at referral and 26.5% (*n* = 373) having three or more. A *χ*^2^ test of independence was performed to examine the relation between presenting difficulties and Community F:CAMHS input. The relation between these variables was significant for anxiety, *χ*^2^ (1, *n* = 1279) = 11.7, *p* < 0.001, with children and young people with anxiety more likely to receive direct input. No other significant differences were found. A *χ*^2^ test of independence was also performed to examine the relation between access to mainstream CAMHS prior to referral and number of presenting difficulties. This was found to be significant, *χ*^2^, *n*** = **1362, = 76.8, df = 4, *p* < 0.001, with children and young people with no prior access to mainstream CAMHS significantly more likely to have fewer presenting difficulties [33.6% (*n* = 118) have 1 presenting difficulty; 22.5% (*n* = 79) have two presenting difficulties; 22.2% (*n* = 78) have three or more presenting difficulties]. Furthermore, children and young people referred to Community F:CAMHS who were currently under the Mental Health Act represented 2.8% of the sample (*n* = 39).

**Table 3:** Mental Health or wellbeing presentation and living conditions at referral, family and personal relationships, and trauma history of children and young people referred to Community F:CAMHS

	All cases		
	Yes	No	Not known/not provided
Presenting difficulties *N* (%)			
Conduct and longstanding behaviour difficulties	674 (47.9)	529 (37.6)	203 (14.4)
Anxiety	502 (35.7)	624 (44.4)	280 (19.9)
ADHD	398 (28.3)	757 (53.8)	251 (17.9)
Autism	372 (26.5)	753 (53.6)	281 (19.9)
Depression	276 (19.6)	827 (58.8)	303 (21.6)
Post-traumatic features	269 (19.1)	799 (56.8)	338 (24.0)
Learning disability	189 (13.3)	931 (66.2)	286 (20.3)
Psychosis	76 (5.4)	1153 (81.9)	178 (12.7)
Other^a^	204 (14.5)	1063 (75.6)	140
Living conditions at referral *N* (%)			
Instability	428 (30.4)	577 (41.0)	401 (28.5)
Absconding/staying away	284 (20.2)	715 (50.9)	407 (28.9)
Short-term/temporary	228 (16.2)	777 (55.3)	401 (28.5)
Offending in family/residential home	212 (15.1)	708 (50.4)	386 (34.8)
Unhealthy/unsafe	179 (12.7)	776 (55.2)	451 (32.1)
Living across multiple residential placements	141 (10.0)	900 (64.0)	365 (26.0)
Living with known offenders	92 (6.5)	832 (59.2)	482 (34.3)
Low-frequency items^b^	76 (5.4)	688 (48.9)	642(45.7)
Family and personal relationships *N* (%)			
Inconsistent supervision/boundary setting	597 (42.5)	365 (26.0)	444 (31.6)
Significant adults failing to show care/interest	432 (30.7)	500 (35.6)	474 (33.7)
Witnessing violence in family context	428 (30.4)	398 (28.3)	580 (41.3)
Experience of abuse	380 (27.0)	476 (33.9)	550 (39.1)
Family/carers involved in criminal activity	364 (25.9)	523 (37.2)	519 (36.9)
Family/carers involved in drug/solvent or alcohol abuse^c^	328 (23.3)	493 (351)	585 (41.6)
Significant bereavement or loss	223 (15.9)	555 (39.5)	629 (44.7)
Low-frequency items^d^	51 (3.6)	446 (31.7)	909 (64.7)
History of trauma *N* (%)			
Neglect/maltreatment	491 (34.9)	448 (31.9)	467 (33.2)
Emotional abuse	466 (33.1)	412 (29.3)	528 (37.6)
Interpersonal violence	431 (30.7)	479 (34.1)	494 (35.3)
Domestic violence	404 (28.7)	444 (31.6)	558 (39.7)
Physical abuse	322 (22.9)	508 (36.1)	576 (41.0)
Community violence	282 (20.1)	584 (41.5)	540 (38.4)
School-based trauma	176 (12.5)	705 (50.1)	525 (37.3)
Sexual abuse	157 (11.2)	613 (43.0)	637 (45.2)
Bereavement	152 (10.8)	698 (49.6)	556 (39.5)
Sexual assault/rape	131 (9.3)	713 (50.7)	562 (40.0)
Low-frequency items^e^	92 (6.5)	613 (43.6)	701 (49.9)

A number of variables with low percentages (< 5%) were combined into ‘low frequency items’ for ease of reporting

^a^‘Other’ presenting difficulties provided by services included for example Foetal Alcohol Syndrome, eating disorders, brain injury, attachment difficulties/disorder, bipolar, developmental delay, emerging personality disorder, emotion dysregulation, gender identity disorder, oppositional defiant, obsessive compulsive disorder, speech and language difficulties, and self-harm

^b^Living conditions at referral: ‘Low-frequency items’ include ‘Over-crowded’ (3.98) and ‘Other’ (1.42)

^c^‘Family/carers involved in drug/solvent or alcohol abuse’ was generated from two variables for ease of reporting: ‘Family/carers involved in drug/solvent abuse’ and ‘Family/carers involved in alcohol abuse’

^d^Family and Personal relationships: ‘Low-frequency items’ include ‘Difficulties with care of his/her own children’ (2.77) and ‘Other’ (0.85)

^e^History of trauma: ‘Low-frequency items’ include ‘War/Political Violence’ (1.14), ‘Serious Accident’ (3.34), ‘Natural Disaster’ (0.07), ‘Terrorism’ (0.57), ‘Kidnapping’ (0.85) and ‘Other’ (1.49)

#### Family and personal relationships

Risks and challenges in family and personal relationships at referral for Community F:CAMHS cases are presented in Table 3. Of children and young people referred, 42.50% (*n* = 597) were known to have experienced inconsistent supervision or boundary setting. Not known/not provided data pertaining to risks and challenges in family or personal relationships ranged between 31.6 and 64.9% (mean = 40.93; See Table 3). In addition, 34.1% (*n* = 479) of children and young people referred to Community F:CAMHS had a family history of mental health difficulties and the families of 37.9% (*n* = 533) had contact with services prior to referral (e.g. mainstream CAMHS, social care).

#### Young people’s history of trauma

Table 3 presents the trauma history of children and young people referred to Community F:CAMHS. 64.2% (*n* = 903) had experienced/witnessed at least one traumatic event and 50.9% (*n* = 715) had experienced/witnessed multiple traumatic events. The most common traumatic events experienced by children and young people referred to Community F:CAMHS were: Neglect or maltreatment (35.0%, *n* = 491), emotional abuse (33.2%, *n* = 466), interpersonal violence (30.7%, *n* = 431), and domestic violence (28.8%, *n* = 404). A *χ*^2^ test of independence was performed to examine the relation between access to mainstream CAMHS prior to referral and trauma history, finding no significant differences between groups.

## Discussion

### Multiplicity of needs and complex presentations

These findings present the first insight into Community F:CAMHS service activity at a national level. They need to be treated with some caution given the proportion of missing data, but they nonetheless confirm the level, range, and scale of the *multiplicity* of diverse needs in this population of children and young people. The multiple and, reciprocally interactive aetiologies of forensic risk in children and young people that these findings demonstrate support previous research findings [13]. Presenting difficulties in the study sample reflect significant and wide-ranging histories of adversity, instability, and trauma. The high levels of family conflict, trauma, and deprivation are consistent with previous findings relating these features to longstanding or reactive attachment difficulties [14], while the relationship between attachment difficulties and the development of mentalizing capacity is well known. Taubner and colleagues [15] recently demonstrated how mentalizing capacity mediates the relationship between early maltreatment and potential for violence in adolescence. The range of young people accessing the services speaks to the flexibility and expertise of Community F:CAMHS in accepting referrals from any service for varied reasons and working with children and young people with a range of presenting difficulties and comorbidities, although youth of colour may be underrepresented (discussed below).

These findings expose the high proportion of early childhood trauma and family or relationship instability present in this cohort of highly complex children and young people, in touch with a range of child services spanning mental health, youth justice and social services. It is evident that there is a need for Community F:CAMHS and other services working with this complex group of children and young people to be trauma-informed and developmentally-attuned. For almost half of the children and young people referred to Community F:CAMHS, Youth Justice Status was not applicable, which highlights the potential for Community F:CAMHS to intervene and provide early support and speaks to the ability of the services to cater for young people both within and outside youth justice settings and processes [6]. In addition, a small number of children and young people in the sample were subject to the Mental Health Act, which suggests there is a legitimate role for Community F:CAMHS in working with secure inpatient providers to support the development of clear, appropriate, and effective future care pathways for this complex group of children and young people. The striking presentation of the extent of *multiplicity* in this population’s needs, which are spread across a broad range of domains (socio-economic, housing/care needs, trauma and abuse, educational exclusion, youth justice, and mental health, etc.) when set alongside the fact that this population is well known to be relatively treatment refractory compared to non-forensic populations, invites further exploration of whether this association (*multiplicity and diversity* of experiences and needs) is causally related, and if so, how.

There is relative over-representation of young people with learning difficulties and neurodevelopmental difficulties (ASD, ADHD) compared to the rates of these conditions in the general population [16, 17] and to the rates of other presenting mental health (MH) difficulties in the study population. This is consistent with literature that disproportionately higher numbers of children and adults with a learning disability or neurodevelopmental difficulties enter the criminal justice system and forensic services [18]. As a nationally specified and commissioned service, Community F:CAMHS operates in and across different Clinical Commissioning Group (CCG) areas. CCGs hold responsibility for the commissioning of local mainstream CAMHS, and as such each CCG area will have different constellations of available services and priority service areas. Similarly, the second highest reason for referral to Community F:CAMHS was for harmful sexual behaviour. The extent to which the over-representation of these diagnostic categories may be accounted for either by relative under-provision in MH services particularly for these groups (due to local commissioning priorities and pressures) or by the specific psychopathologies captured in these diagnoses themselves (these being more likely to lead the young person into boundary infringements) is unanswered and remains a key consideration for policy and commissioning when aiming for parity of provision.

### Demographic characteristics

Categorisations of ethnicity and gender in this study used NHS classifications. A key finding is the observation of a distinct gender split, with significant differences found between key characteristics for girls and boys such as referral source, social care status and reasons for referral including youth justice. More girls were referred by mental health services while more boys were referred by or because of the youth justice system, despite exhibiting similar concerning behaviours. Previous research has suggested that female adolescents report higher levels of internalising symptoms [19, 20], which may help explain the differing referral pathways. Furthermore, a higher proportion of girls in the current sample (compared to boys) were in the care of the state, either as Looked After Children or in youth custody. This raises concerns for this group and may highlight a need for more effective early intervention for girls in the community who pose a professional concern. What is also evident in the sample is that many children and young people referred to Community F:CAMHS are not subject to criminal justice interventions. This underlines the fact that many children and young people present with high risk of harm to others without involvement in the criminal justice system and highlights the need for services such as Community F:CAMHS in such cases. There were also a number of children in the sample who were aged under 11 years; whilst this may be surprising to some, it is in line with longitudinal studies which identify some children with severe behaviour difficulties from an early age [21].

The issue of racial and ethnic disparities in mental health and specifically how Black, Asian and minoritised ethnic groups access Community F:CAMHS is a significant finding. There is an apparent over-representation of White or White British children and young people receiving input from Community F:CAMHS when compared to the known over-representation of children and young people from minoritised ethnic backgrounds in the criminal justice system [22], as well as the diverse ethnic makeup of geographical regions, namely in major cities. Contact with state institutions can be a source of (re)traumatisation for minoritised ethnic groups who often experience disproportionate exclusion through a failure to provide culturally sensitive services. National data for mainstream CAMHS shows clearly that, as a proportion of the population, children and young people from minoritised ethnic backgrounds are under-represented in accessing and using these services [23–25]. It is known that Black and Asian young people are over-represented in criminal justice pathways and underrepresented in mental health pathways. Minoritised ethnic people are also more likely to access mental health services through criminal justice pathways than their White counterparts [26]. There is a specific remit here for Community F:CAMHS to ensure they are meeting the mental health needs of minoritised ethnic young people in the community.

It is crucial that Community F:CAMHS is accessible to those children and young people historically and presently underserved, including those from minoritised ethnic backgrounds, with the aim of supporting early intervention and reducing the risk of criminalisation in the context of mental health difficulties. There is cause for further investigation about the accessibility and sensitivity of Community F:CAMHS to identifying and assessing needs in all groups, perhaps as result of wider challenges in accessing and using services which would typically refer. It is possible that Community F:CAMHS teams are not consistently representing the local populations in which they operate, as the data from this study shows that 70% of children and young people have been referred to Community F:CAMHS from a mainstream CAMHS team. There are many possible other explanations that the data presented here cannot elucidate and future research is required to explore potential referrer bias as a matter of priority to ensure equitable access and provision.

The Lammy Review [22], which calls for wide reforms of the criminal justice system, records the treatment and experience of people from minoritised ethnic groups and makes a number of recommendations. The recommendations are useful to consider these results against. Recommendation 2 notes the differing system of recording ethnicity in the US and suggests that datasets such as these should be interrogated biannually; the current regime of ethnicity data collection for Community F:CAMHS would appear to be sufficient but there is room to consider action in order to increase sensitivity as part of improving the equity of reach for the services. Recommendation 4 of the Lammy Review calls for a principle of ‘explain or reform’ and we would suggest that adopting such an approach would address the narrative of the above data.

### Multi-agency working

There is an unanswered question as to whether *iatrogenesis* (perhaps in the form of repeated, differentially-targeted, duplicated and dis-integrated brief interventions over time from multiple agencies and professionals from health, social care, education and youth justice, etc.) may contribute to the challenging equifinality of outcomes in this population. Further investigation into past experiences of ‘help’ in this population deemed ‘high risk’ suggests that professional help may repeatedly be experienced as *unhelpful* (and henceforth prematurely rejected) precisely because it tends to be delivered via multiple short-term relationships with multiple workers/agencies whose offerings are experienced as fragmented and dis-integrated (an industrialised “doing to” rather than “with”) [27, 28]. The disjuncture between children and young people and the professionals with whom they interact, paired with experiencing care as unhelpful or disjointed, may result in the additional challenge of a child or young person failing to develop ‘epistemic trust’, the necessary precursor to effective therapeutic engagement, with these and future people offering help.

It has been proposed that this ‘epistemic hypervigilance’ may result in chronicity and difficulties independent of specific diagnosis or symptom cluster or severity at a given point in time [29]. Further research is required to explore the impact of Community F:CAMHS involvement on children and young people’s outcomes and exploring predictors of outcomes within the sample to help inform service development and provision. Furthermore, future research could conduct pathway analysis to explore routes to Community F:CAMHS involvement and provide important information on children and young people’s journeys through services. Qualitative exploration of the experiences of children and young people, and their parents or guardians, would shed light on individual experiences of relationships with professionals and of being in contact with a network of services.

It is possible that the involvement of Community F:CAMHS supports mitigation against harm, equipping existing services to better serve children and young people, providing continuity of care and filling gaps in expertise and service provision. This hypothesis may be supported by the data looking at children and young people referred who had previously accessed mainstream CAMHS versus those who had not. Over a quarter of young people referred to Community F:CAMHS in our sample had not been in contact with mainstream CAMHS previously. This corroborates previous findings of high-risk young people often not coming into contact with mainstream CAMHS despite high proportions of young people in forensic services having unmet mental health needs [10, 30]. Indeed, it is has been suggested that mainstream CAMHS staff may be ambivalent about accepting referrals for children and young people presenting with behavioural or conduct difficulties [31]. This finding may also be a result of the mainstream CAMHS service model, which may lack the flexibility and cultural sensitivity necessary to work with some groups. These are likely significant factors contributing to the known difficulties in help-seeking experienced by certain communities, for example children and young people from minoritised ethnic backgrounds, or those in contact with welfare or youth justice services [25, 32, 33]. However, comparative analysis in our sample showed that children and young people who had not accessed mainstream CAMHS prior to referral in our sample had significantly fewer presenting difficulties and were more likely to receive indirect Community F:CAMHS input. This may suggest that Community F:CAMHS do not operate as an ‘additional’ service, and speaks to their specialism and low threshold for referrals. Furthermore, the data shows that Community F:CAMHS receive a significant proportion of referrals from non-health settings (e.g. Social Care or Youth Offending Teams), which offers hope towards addressing gaps in services identified by Hindley and colleagues [6] in integrating forensic services for young people with mental health difficulties into overall care pathways for all young people. Importantly, there remains a risk of Community F:CAMHS undertaking work which falls into the remit of other services, which may impact on the sustainability of Community F:CAMHS.

### Strengths and limitations

Some limitations of the current research include using service activity data for secondary analysis, thus accepting that records can be subject to selection bias, confounding and missing data [34]. Clinical accounts may also be influenced by the subjective opinion of the clinician providing the data, and it is not known whether any of the data used was self-reported. Furthermore, as data were collected and submitted by services, there is a risk of inconsistent data reporting across services, as well as a high potential for missing data, as seen in the results. Demographic characteristics including gender and ethnicity were based on NHS and Office for National Statistics categories for consistency with services data systems. These categorisations are conceptually limited in their scope and precision and may not reflect how young people identify themselves, which could have serious implications for how young people are treated in their contact with services.

Future research should consider more inclusive forms of demographic data collection such as self-reported free text options to understand more accurately the experiences of subgroups within the population. The data were also collected as part of a wider national evaluation, and it is possible that the data submitted show a favourable perspective of Community F:CAMHS activity due to social desirability bias. The data were collected over a relatively short timeframe and may not capture those cases which require longer-term Community F:CAMHS input. Future research with this population of children and young people may shed light on the gaps in and across networks and inform future policy and practice. Notwithstanding the above limitations, a major strength of the current research is its contribution to the limited evidence-base on Community F:CAMHS service activity and of the characteristics of children and young people who access these specialist services. Furthermore, the use of service activity data for observational research also has advantages. The naturalistic approach of the study provides good external and ecological validity, reducing information bias (e.g. recall bias).

This research provides the first national description of children and young people referred to Community F:CAMHS in England, confirming the level, range, and scale of the multiplicity of diverse needs in this population and the high levels of significant and wide-ranging histories of adversity, instability, and trauma. The findings additionally provide insight into service activity and provision, indicating the range of services involved in this cohort’s care and identifying potential gaps in services. For example, our results show that a quarter of referrals had not previously accessed mainstream CAMHS and demonstrate the relative over-representation of children and young people with learning difficulties & neurodevelopmental difficulties or exhibiting harmful sexual behaviours in contact with Community F:CAMHS. The data corroborate previous research highlighting the complexities and vulnerabilities of the group, and the need for interagency trauma-informed working. Further research is required to examine the impact of Community F:CAMHS on children and young people’s outcomes and to better understand pathways through services.

## Supplementary Information

Below is the link to the electronic supplementary material.

Supplementary file1 (DOCX 49 kb)

## Data Availability

The data are available upon appropriate request to CORC@annafreud.org subject to approval by NHS England and NHS Improvement.
