# Supplementary material for: Characteristics of young people accessing recently implemented Community Forensic Child and Adolescent Mental Health Services (F:CAMHS) in England: insights from national service activity data
Source: Eur Child Adolesc Psychiatry. 2021 Sep 14;32(3):405–17. doi: 10.1007/s00787-021-01870-y (PMC10038947; doi:10.1007/s00787-021-01870-y)
Supplement: Supplementary file 1 — Supplementary file1 (DOCX 49 kb) [file 787_2021_1870_MOESM1_ESM.docx]

**Appendix - Supplementary materials**

**Supplementary Table 1.** Characteristics and service activity broken down by young people with/ without prior CAMHS contact.

|  | **Prior CAMHS contact** | |
| --- | --- | --- |
|  | **No (N =351)** | **Yes (N=1011)** |
| **Age***** |  |  |
| <=8 | 24 (6.84) | 14 (1.38) |
| 9 -11 | 44 (12.5) | 68 (6.73) |
| 12-15 | 183 (52.1) | 531 (52.5) |
| 16+ | 100 (28.5) | 398 (39.4) |
| Missing/ not provided | 0 (0) | 0 (0) |
| **Gender**** |  |  |
| Female (including trans-female) | 43 (12.3) | 191 (18.9) |
| Male (including trans-male) | 307 (87.5) | 819 (81.0) |
| Missing/ not provided | SN^a^ | SN |
| **Ethnicity** |  |  |
| Asian/Asian British | 10 (2.85) | 28 (2.77) |
| Black/Black British | 25 (7.12) | 45 (4.45) |
| Mixed | 33 (9.40) | 87 (8.61) |
| White | 256 (72.9) | 802 (79.3) |
| Other ethnic group | 9 (2.56) | 19 (1.88) |
| Not known/ not provided | 18 (5.13) | 30 (2.97) |
| **Referral Source***** |  |  |
| Mainstream CAMHS | 83 (23.6) | 521 (51.5) |
| Education | 41 (11.7) | 45 (4.45) |
| Social care | 125 (35.6) | 239 (23.6) |
| Youth justice | 71 (20.2) | 136 (13.5) |
| Other health | 15 (4.27) | 41 (4.06) |
| Third sector | 3 (0.85) | 5 (0.49) |
| GP | 7 (1.99) | 6 (0.59) |
| Other | 6 (1.71) | SN |
| Not known/ not provided | 0 (0) | SN |
| **Contact type*** |  |  |
| Indirect | 233 (66.4) | 671 (66.4) |
| Direct | 86 (24.5) | 278 (27.5) |
| Rejected – not appropriate | 24 (6.84) | 32 (3.17) |
| Rejected – out of area | 0 (0) | 8 (0.79) |
| Other | 7 (1.99) | 20 (1.98) |
| Missing/ not provided | SN | SN |
| **Social care status*** |  |  |
| Social Care or Early Help Plan | 138 (39.3) | 361 (35.7) |
| Looked After Children | 82 (23.4) | 337 (33.3) |
| No Social Care Involvement | 98 (27.9) | 228 (22.6) |
| Other | 29 (8.26) | 78 (7.72) |
| Not known/ not provided | 4 (1.14) | 7 (0.69) |
| **Youth justice status** |  |  |
| Recent police contact | 86 (24.5) | 232 (22.9) |
| Pre-sentencing | 26 (7.41) | 85 (8.41) |
| Sentenced | 50 (14.2) | 138 (13.6) |
| Other | 32 (9.12) | 51 (5.04) |
| Not applicable | 155 (44.2) | 500 (49.5) |
| Not known/ not provided | SN | 5 (0.50) |
| **Reason for referral** |  |  |
| *Violence and aggression **** |  |  |
| Yes | 267 (76.1) | 855 (84.6) |
| No | 75 (21.4) | 139 (13.7) |
| Not known/ not provided | 9 (2.56) | 17 (1.68) |
| *Sexually harmful behaviour* |  |  |
| Yes | 111 (31.6) | 312 (30.9) |
| No | 231 (65.8) | 682 (67.5) |
| Missing/ not known | 9 (2.56) | 17 (1.68) |
| *Second opinion in a complex case**** |  |  |
| Yes | 79 (22.5) | 354 (35.0) |
| No | 263 (74.9) | 638 (63.1) |
| Missing/ not provided | 9 (2.56) | 19 (1.88) |
| *Fire setting* |  |  |
| Yes | 37 (10.5) | 110 (10.9) |
| No | 305 (86.9) | 883 (87.3) |
| Missing/ not provided | 9 (2.56) | 18 (1.78) |
| *Youth justice* |  |  |
| Yes | 92 (26.2) | 256 (25.3) |
| No | 250 (71.2) | 736 (72.8) |
| Missing/ not provided | 9 (2.56) | 19 (1.88) |
| *Other* |  |  |
| Yes | 62 (17.7) | 171 (16.9) |
| No | 275 (78.3) | 815 (80.6) |
| Missing/ not provided | 14 (3.99) | 25 (2.47) |
| **Presenting difficulties** |  |  |
| *Psychosis ** |  |  |
| Yes | 9 (2.56) | 66 (6.53) |
| No | 299 (85.2) | 851 (84.2) |
| Missing/ not provided | 43 (12.3) | 94 (9.30) |
| *Anxiety ** |  |  |
| Yes | 103 (29.3) | 398 (39.4) |
| No | 167 (47.6) | 456 (45.1) |
| Missing/ not provided | 81 (23.1) | 157 (15.5) |
| *Depression*** |  |  |
| Yes | 49 (14.0) | 227 (22.5) |
| No | 224 (63.8) | 602 (59.5) |
| Missing/ not provided | 78 (22.2) | 182 (18.0) |
| *Post traumatic features* |  |  |
| Yes | 55 (15.7) | 214 (21.2) |
| No | 203 (57.8) | 595 (58.9) |
| Missing/ not provided | 93 (26.5) | 202 (20.0) |
| *ADHD**** |  |  |
| Yes | 61 (17.4) | 336 (33.2) |
| No | 207 (59.0) | 549 (54.3) |
| Missing/ not provided | 83 (23.6) | 126 (12.5) |
| *Autism**** |  |  |
| Yes | 52 (14.8) | 320 (31.7) |
| No | 200 (57.0) | 552 (54.6) |
| Missing/ not provided | 99 (28.2) | 139 (13.7) |
| *Conduct and longstanding behaviour disorders** |  |  |
| Yes | 141 (40.2) | 530 (52.4) |
| No | 144 (41.0) | 385 (38.1) |
| Missing/ not provided | 66 (18.8) | 96 (9.50) |
| *Learning disability* |  |  |
| Yes | 40 (11.4) | 149 (14.7) |
| No | 241 (68.7) | 689 (68.2) |
| Missing/ not provided | 70 (19.9) | 173 (17.1) |
| *Other* |  |  |
| Yes | 47 (13.4) | 156 (15.4) |
| No | 272 (77.5) | 789 (78.0) |
| Missing/ not provided | 32 (9.12) | 66 (6.53) |
| **Number of presenting difficulties ***** |  |  |
| None | 54 (15.4) | 79 (7.81) |
| 1 | 118 (33.6) | 196 (19.4) |
| 2 | 79 (22.5) | 252 (24.9) |
| 3+ | 78 (22.2) | 449 (44.4) |
| Missing/ not provided | 22 (6.27) | 35 (3.46) |
| **Number of trauma histories** |  |  |
| None | 62 (17.7) | 137 (13.6) |
| 1 | 51 (14.5) | 137 (13.6) |
| 2 | 43 (12.3) | 120 (11.9) |
| 3+ | 133 (37.9) | 417 (41.2) |
| Missing/ not provided | 62 (17.7) | 200 (19.8) |

N=1,362 with CAMHS prior contact yes/no provided. Chi-squared tests were performed to compare characteristics and service activity between young people with/ without prior CAMHS contact - * p<0.05, ** p<0.01,*** p<0.001. Missing/ not provided category was not included in Chi-squared tests for ‘Reason for referral’ and ‘Presenting difficulties’ only. ^a^SN- small numbers (< 3) are not reported to protect anonymity, data may also not be reported to prevent calculation of small numbers.

**Supplementary Table 2.** Characteristics and service activity broken down by gender

|  | **Gender** | |
| --- | --- | --- |
|  | **Male (including trans male)**  **(N =1160)** | **Female (including trans female)**  **(N =238)** |
|  | **N (%)** | **N(%)** |
| **Age*** |  |  |
| <=8 | 36 (3.10) | SN^a^ |
| 9 -11 | 102 (8.79) | 12 (5.04) |
| 12-15 | 590 (50.9) | 142 (59.7) |
| 16+ | 431 (37.2) | 82 (34.5) |
| Missing/ not provided | SN | SN |
| **Ethnicity** |  |  |
| Asian/Asian British | 34 (2.93) | 6 (2.52) |
| Black/Black British | 65 (5.60) | 12 (5.04) |
| Mixed | 99 (8.53) | 24 (10.1) |
| White | 897 (77.3) | 184 (77.3) |
| Other ethnic group | 26 (2.24) | 3 (1.26) |
| Not known/ not provided | 39 (3.36) | 9 (3.78) |
| **Referral Source**** |  |  |
| Mainstream CAMHS | 478 (41.2) | 126 (52.9) |
| Education | 80 (6.90) | 6 (2.52) |
| Social Care | 299 (25.8) | 66 (27.7) |
| Youth justice | 188 (16.2) | 22 (9.24) |
| Other Health | 47 (4.05) | 9 (3.78) |
| Third Sector | 7 (0.60) | SN |
| GP | 13 (1.12) | 0 (0) |
| Other | 20 (1.72) | 4 (1.68) |
| Not known/ not provided | 28 (2.41) | SN |
| **Contact type** |  |  |
| Indirect | 754 (65.0) | 161 (67.6) |
| Direct | 301 (25.9) | 62 (26.1) |
| Rejected – not appropriate | 52 (4.48) | 5 (2.10) |
| Rejected – out of area | 7 (0.60) | SN |
| Other | 21 (1.81) | 6 (2.52) |
| Missing/ not provided | 25 (2.16) | SN |
| **Social care status***** |  |  |
| Social Care or Early Help Plan | 426 (36.7) | 73 (30.7) |
| Looked After Children | 320 (27.6) | 103 (43.3) |
| No Social Care Involvement | 280 (24.1) | 44 (18.5) |
| Other | 95 (8.91) | 13 (5.46) |
| Not known/ not provided | 39 (3.36) | 5 (2.10) |
| **Youth justice status** |  |  |
| Recent police contact | 268 (23.1) | 12 (5.04) |
| Pre-sentencing | 100 (8.62) | 50 (21.0) |
| Sentenced | 164 (14.1) | 26 (10.9) |
| Other | 71 (6.12) | 13 (5.46) |
| Not applicable | 524 (45.2) | 131 (55.0) |
| Not known/ not provided | 33 (2.84) | 6 (2.52) |
| **Reason for referral** |  |  |
| *Violence and aggression *** |  |  |
| Yes | 918 (79.1) | 205 (86.1) |
| No | 192 (16.6) | 23 (9.66) |
| Not known/ not provided | 50 (4.31) | 10 (4.20) |
| *Sexually harmful behaviour**** |  |  |
| Yes | 388 (33.4) | 35 (14.7) |
| No | 722 (62.2) | 193 (81.1) |
| Not known/ not provided | 50 (4.31) | 10 (4.20) |
| *Second opinion in a complex case* |  |  |
| Yes | 356 (30.7) | 77 (32.4) |
| No | 753 (64.9) | 150 (63.0) |
| Not known/ not provided | 51 (4.40) | 11 (4.62) |
| *Fire setting* |  |  |
| Yes | 124 (10.7) | 23 (9.66) |
| No | 986 (85.0) | 204 (85.7) |
| Not known/ not provided | 50 (4.31) | 11 (4.62) |
| *Youth justice*** |  |  |
| Yes | 308 (26.6) | 40 (16.8) |
| No | 801 (69.1) | 187 (78.6) |
| Not known/ not provided | 51 (4.40) | 11 (4.62) |
| *Other* |  |  |
| Yes | 190 (16.4) | 43 (18.1) |
| No | 909 (78.4) | 183 (76.9) |
| Not known/ not provided | 61 (5.26) | 12 (5.04) |
| **Presenting difficulties** |  |  |
| *Psychosis* |  |  |
| Yes | 61 (5.26) | 14 (5.88) |
| No | 958 (82.6) | 193 (81.1) |
| Not known/ not provided | 141 (12.2) | 31 (13.0) |
| *Anxiety** |  |  |
| Yes | 399 (34.4) | 103 (43.3) |
| No | 527 (45.4) | 95 (39.9) |
| Not known/ not provided | 234 (20.2) | 40 (16.8) |
| *Depression**** |  |  |
| Yes | 202 (17.4) | 74 (31.1) |
| No | 705 (60.8) | 120 (50.4) |
| Not known/ not provided | 253 (21.8) | 44 (18.5) |
| *Post traumatic features**** |  |  |
| Yes | 192 (16.6) | 77 (32.4) |
| No | 675 (58.2) | 122 (51.3) |
| Not known/ not provided | 293 (25.3) | 39 (16.4) |
| *ADHD**** |  |  |
| Yes | 350 (30.2) | 47 (19.7) |
| No | 605 (52.2) | 151 (63.4) |
| Not known/ not provided | 205 (17.7) | 40 (16.8) |
| *Autism* |  |  |
| Yes | 310 (26.7) | 62 (26.1) |
| No | 610 (52.6) | 141 (59.2) |
| Not known/ not provided | 240 (20.7) | 35 (14.7) |
| *Conduct and longstanding behaviour disorders* |  |  |
| Yes | 556 (47.9) | 118 (49.6) |
| No | 431 (37.2) | 96 (40.3) |
| Not known/ not provided | 173 (14.9) | 24 (10.1) |
| *Learning disability* |  |  |
| Yes | 161 (13.9) | 28 (11.8) |
| No | 766 (66.0) | 163 (68.5) |
| Not known/ not provided | 233 (20.1) | 47 (19.7) |
| *Other*** |  |  |
| Yes | 152 (13.1) | 51 (21.4) |
| No | 894 (77.1) | 167 (70.2) |
| Not known/ not provided | 114 (9.83) | 20 (8.40) |
| Number of presenting difficulties* |  |  |
| None | 117 (10.1) | 16 (6.72) |
| 1 | 261 (22.5) | 52 (21.8) |
| 2 | 285 (24.6) | 47 (19.7) |
| 3+ | 417 (35.9) | 111 (46.6) |
| Not known/ not provided | 80 (6.90) | 12 (5.04) |
| Number of trauma histories |  |  |
| None | 174 (15.0) | 25 (10.5) |
| 1 | 158 (13.6) | 30 (12.6) |
| 2 | 142 (12.2) | 22 (9.24) |
| 3+ | 440 (36.2) | 111 (46.6) |
| Not known/ not provided | 246 (21.2) | 50 (21.0) |

N=1,398 with gender data provided. Chi-squared tests were performed to compare characteristics and service activity between gender- * p<0.05, ** p<0.01,*** p<0.001. Missing/ not provided data was not included in Chi-squared tests for reason for referral and presenting difficulties only. ^a^SN- small numbers (< 3) are not reported to protect anonymity, data may also not reported to prevent calculation of small numbers

**Supplementary Table 3.** Characteristics broken down by case type

|  | **Case type** |  |
| --- | --- | --- |
|  | **Direct**  **N = 364** | **Indirect**  **N = 915** |
|  | **N (%)** | **N (%)** |
| **Age*** |  |  |
| <=8 | 3 (0.82) | 30 (3.28) |
| 9 -11 | 21 (5.77) | 85 (9.29) |
| 12-15 | 196 (53.8) | 475 (51.9) |
| 16+ | 144 (39.6) | 325 (35.5) |
| Not known/ not provided | 0 (0) | 0 (0) |
| **Ethnicity** |  |  |
| Asian/Asian British | 12 (3.30) | 25 (2.73) |
| Black/Black British | 18 (4.95) | 48 (5.25) |
| Mixed | 31 (8.52) | 77 (8.42) |
| White | 286 (78.6) | 720 (78.7) |
| Other ethnic group | 4 (1.10) | 22 (2.40) |
| Not known/ not provided | 13 (3.57) | 23 (2.51) |
| **Referral Source*** |  |  |
| Mainstream CAMHS | 183 (50.3) | 391 (42.7) |
| Education | 16 (4.40) | 64 (6.99) |
| Social Care | 80 (22.0) | 253 (27.7) |
| Youth justice | 56 (15.4) | 139 (15.2) |
| Other Health | 16 (4.40) | 35 (3.83) |
| Third Sector | SN^a^ | 6 (0.66) |
| GP | 6 (1.65) | 4 (0.44) |
| Other | SN | 16 (1.75) |
| Not known/ not provided | 0 (0) | 7 (0.77) |
| **Social care status** |  |  |
| Social Care or Early Help Plan | 139 (38.2) | 329 (36.0) |
| Looked After Children | 97 (26.6) | 299 (32.7) |
| No Social Care Involvement | 97 (26.6) | 208 (22.7) |
| Other | 29 (7.97) | 65 (7.10) |
| Not known/ not provided | SN | 14 (1.53) |
| **Youth justice status** |  |  |
| Recent police contact | 100 (27.5) | 199 (21.7) |
| Pre-sentencing | 23 (6.32) | 84 (9.18) |
| Sentenced | 49 (13.5) | 129 (14.1) |
| Other | 23 (6.32) | 56 (6.12) |
| Not applicable | 167 (45.9) | 436 (47.7) |
| Not known/ not provided | SN | 11 (1.20) |
| **Reason for referral** |  |  |
| *Violence and aggression* |  |  |
| Yes | 303 (83.2) | 744 (81.3) |
| No | 60 (16.5) | 138 (15.1) |
| Not known/ not provided | SN | 33 (3.61) |
| *Sexually harmful behaviour* |  |  |
| Yes | 121 (33.2) | 271 (29.6) |
| No | 242 (66.5) | 611 (66.8) |
| Not known/ not provided | SN | 33 (3.61) |
| *Second opinion in a complex case*** |  |  |
| Yes | 96 (26.4) | 317 (34.6) |
| No | 267 (73.4) | 563 (61.5) |
| Not known/ not provided | SN | 35 (3.83) |
| *Fire setting* |  |  |
| Yes | 37 (10.2) | 103 (11.3) |
| No | 326 (89.6) | 778 (85.0) |
| Not known/ not provided | SN | 34 (3.72) |
| *Youth justice** |  |  |
| Yes | 115 (31.6) | 223 (24.4) |
| No | 248 (68.1) | 657 (71.8) |
| Not known/ not provided | SN | 35 (3.83) |
| *Other* |  |  |
| Yes | 58 (15.9) | 149 (16.3) |
| No | 305 (83.8) | 720 (78.7) |
| Not known/ not provided | SN | 46 (5.03) |
| **Presenting difficulties** |  |  |
| *Psychosis* |  |  |
| Yes | 21 (5.77) | 54 (5.90) |
| No | 315 (86.5) | 755 (82.5) |
| Not known/ not provided | 28 (7.69) | 106 (11.6) |
| *Anxiety**** |  |  |
| Yes | 168 (46.2) | 306 (33.4) |
| No | 147 (40.4) | 429 (46.9) |
| Not known/ not provided | 49 (13.5) | 180 (19.7) |
| *Depression* |  |  |
| Yes | 81 (22.3) | 175 (19.1) |
| No | 234 (64.3) | 538 (58.8) |
| Not known/ not provided | 49 (13.5) | 202 (22.1) |
| *Post traumatic features* |  |  |
| Yes | 81 (22.3) | 173 (18.9) |
| No | 212 (58.2) | 525 (57.4) |
| Not known/ not provided | 71 (19.5) | 217 (23.7) |
| *ADHD* |  |  |
| Yes | 110 (30.2) | 266 (29.1) |
| No | 208 (57.1) | 495 (54.1) |
| Not known/ not provided | 46 (12.6) | 154 (16.8) |
| *Autism* |  |  |
| Yes | 115 (31.6) | 239 (26.1) |
| No | 189 (51.9) | 506 (55.3) |
| Not known/ not provided | 60 (16.5) | 170 (18.6) |
| *Conduct and longstanding behaviour disorders* |  |  |
| Yes | 211 (58.0) | 436 (47.7) |
| No | 130 (35.7) | 348 (38.0) |
| Not known/ not provided | 23 (6.32) | 131 (14.3) |
| *Learning disability* |  |  |
| Yes | 42 (11.5) | 135 (14.8) |
| No | 269 (73.9) | 595 (65.0) |
| Not known/ not provided | 53 (14.6) | 185 (20.2) |
| *Other* |  |  |
| Yes | 51 (14.0) | 132 (14.4) |
| No | 296 (81.3) | 705 (77.0) |
| Not known/ not provided | 17 (4.67) | 78 (8.52) |
| **Number of presenting difficulties***** |  |  |
| None | 18 (4.95) | 100 (10.9) |
| 1 | 69 (19.0) | 216 (23.6) |
| 2 | 100 (27.5) | 211 (23.1) |
| 3+ | 166 (45.6) | 339 (37.0) |
| Not known/ not provided | 11 (3.02) | 49 (5.36) |
| **Number of trauma histories***** |  |  |
| None | 50 (13.7) | 125 (13.7) |
| 1 | 52 (14.3) | 124 (13.6) |
| 2 | 48 (13.2) | 107 (11.7) |
| 3+ | 170 (46.7) | 355 (38.8) |
| Not known/ not provided | 44 (12.1) | 204 (22.3) |

N=1,279 cases with indirect or direct contact. Chi-squared tests were performed to compare characteristics and service activity between gender- * p<0.05, ** p<0.01,*** p<0.001. Missing/ not provided data was not included in Chi-squared tests for reason for referral and presenting difficulties only. ^a^SN- small numbers (< 3) are not reported to protect anonymity, data may also not reported to prevent calculation of small numbers.
